# Topography of emotional valence and arousal within the motor part of the subthalamic nucleus in Parkinson’s disease

**DOI:** 10.1038/s41598-019-56260-x

**Published:** 2019-12-27

**Authors:** Tereza Serranová, Tomáš Sieger, Filip Růžička, Eduard Bakštein, Petr Dušek, Pavel Vostatek, Daniel Novák, Evžen Růžička, Dušan Urgošík, Robert Jech

**Affiliations:** 1 Department of Neurology and Center of Clinical Neuroscience, Charles University, 1st Faculty of Medicine and General University Hospital, Kateřinská 30, 128 08 Prague, Czech Republic; 2 Department of Cybernetics, Faculty of Electrical Engineering, Czech Technical University in Prague, Technická 2, 166 27 Prague, Czech Republic; 3 Department of Stereotactic and Radiation Neurosurgery, Na Homolce Hospital, Roentgenova 2, 150 30 Prague, Czech Republic; 4 National Institute of Mental Health, Klecany, Topolová 748, 250 67 Czech Republic

**Keywords:** Limbic system, Parkinson's disease

## Abstract

Clinical motor and non-motor effects of deep brain stimulation (DBS) of the subthalamic nucleus (STN) in Parkinson's disease (PD) seem to depend on the stimulation site within the STN. We analysed the effects of the position of the stimulation electrode within the motor STN on subjective emotional experience, expressed as emotional valence and arousal ratings to pictures representing primary rewards and aversive fearful stimuli in 20 PD patients. Patients’ ratings from both aversive and erotic stimuli matched the mean ratings from a group of 20 control subjects at similar position within the STN. Patients with electrodes located more posteriorly reported both valence and arousal ratings from both the rewarding and aversive pictures as more extreme. Moreover, posterior electrode positions were associated with a higher occurrence of depression at a long-term follow-up. This brain–behavior relationship suggests a complex emotion topography in the motor part of the STN. Both valence and arousal representations overlapped and were uniformly arranged anterior-posteriorly in a gradient-like manner, suggesting a specific spatial organization needed for the coding of the motivational salience of the stimuli. This finding is relevant for our understanding of neuropsychiatric side effects in STN DBS and potentially for optimal electrode placement.

## Introduction

Perception and experience of emotion in humans seem to be implemented by a range of cortical and subcortical regions involved in large-scale brain networks according to evidence from numerous neuroimaging studies^1,2^. However, neuroimaging methods are inherently limited by low spatial and temporal resolution in comparison with invasive techniques recording local field potentials or single-unit activity. Since the opportunities to use such techniques in humans are rare, mapping of neural representation of emotion on a small-scale remains highly fragmented.

Deep brain stimulation (DBS) of the subthalamic nucleus (STN), which has become a standard treatment for motor complications in Parkinson's disease (PD)^3,4^ has provided a unique opportunity to study the functional architecture of the basal ganglia in humans. Besides motor control and action selection, the basal ganglia have an important role in decision-making, emotion, language, learning, memory, and more^5^. Current knowledge of the functional organization of frontal cortico-basal ganglia network highlights the integration of information from functionally distinct cortical regions in the target basal ganglia (i.e. the striatum and the STN) established by a convergence of cortical projections to create zones of unique combinations of afferents^6–13^. These, in turn, may have unique functions^7^.

In line with findings of functional overlap of the dorsolateral motor, central associative, and ventromedial limbic territory of the STN^14^, we recently identified single-neurons responding to emotional stimuli in the dorsolateral motor territory of the STN in PD patients undergoing STN DBS^15^. At the same time, clinical motor and non-motor effects of DBS seem to depend on the electrode position within the STN in a manner that reflects the functional organization and connectivity of the STN^16–19^. Surprisingly, despite the high rate of neuropsychiatric side effects and changes in emotional processes found in patients successfully treated by STN DBS for motor complications^20^, the effect of the electrode position on subjective emotional experience has not been studied so far.

Emotional behaviour is organized along two psycho-physiological dimensions: emotional valence, varying from negative to neutral to positive, and arousal, varying from low to high^21,22^. Previously, we found the effects of the STN DBS on subjective valence and arousal ratings from affective pictures representing primary rewards and aversive fearful stimuli. With the DBS switched ON, aversive fearful stimuli were discerned as more negative according to their valence ratings^23^.

Considering the functional anatomy of the STN, in this study we further analysed the relationship between the electrode position within the STN and individual affective ratings in PD patients treated with bilateral STN DBS. Emotional valence and arousal ratings from pictures representing primary rewards (visual sexual stimuli) and aversive fearful stimuli were recorded postoperatively in OFF-medicated PD patients with the stimulation ON and OFF and in matched control subjects. Coordinates of each active electrode contact and anatomical delineation of each individual STN were determined from a pre- and postoperative MRI and averaged from both hemispheres. Each STN was transformed into the standardized stereotactic space (Montreal Neurological Institute – MNI)^24^ and both the active electrode contacts and the positions of the neurons responding to either emotional valence or arousal from our previous study were plotted together to compare the functional anatomy of emotional valence and arousal on large and small scales.

## Results

### Electrode position and affective ratings

**Figure 1:**
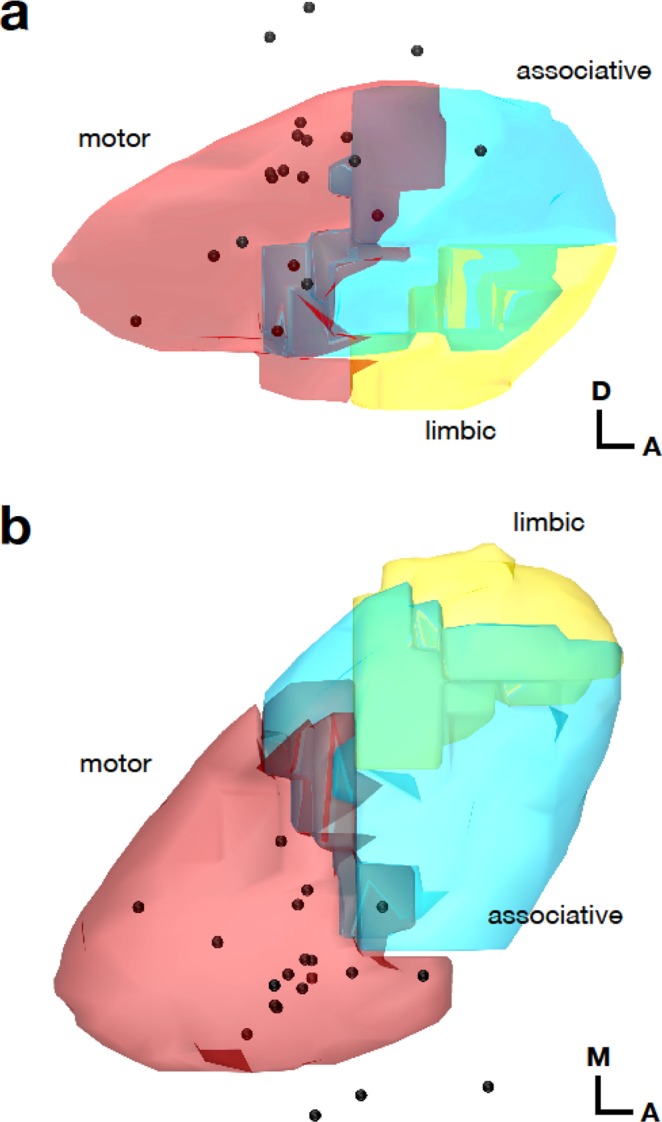
Positions of the active DBS electrode contacts. (**a**) Sagittal and (**b**) axial view of the common active DBS electrode contacts in the context of the motor, associative, and limbic sub-territories of the right STN^70^. As each patient was stimulated bilaterally, the common active electrode contact was defined as the point lying halfway between the right active electrode contact and the left active electrode contact mirrored to the right hemisphere. Note that most contacts lay within the motor region of the STN.

The positions of the active electrode contacts within the STN plotted in the standardized stereotactic MNI space with the delineation of the motor, associative and limbic region are shown in Fig. 1. The position of the active electrode contacts within the STN varied from y = −16.5 mm to y = −10.7 in antero-posterior direction, from x = 11.0 mm to x = 15.5 mm in medio-lateral direction, and from z = −9.7 to −4.3 mm in dorso-ventral direction. However, only the positions along the antero-posterior axis were associated with a gradient-like organization reflecting emotional ratings.

**Figure 2:**
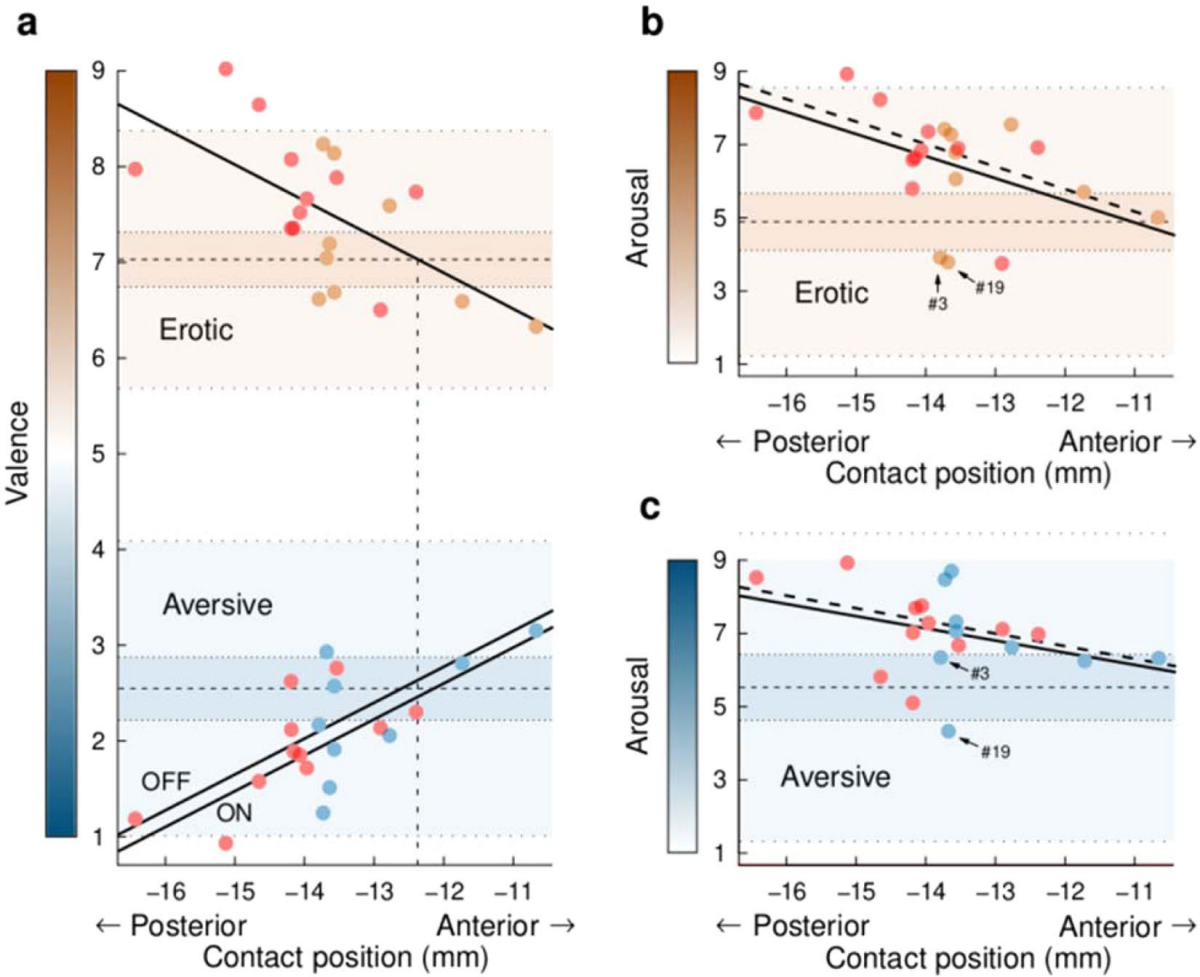
The gradients of emotional ratings in respect to the antero-posterior position of the active electrode contact. **(a)** Valence ratings of erotic (top) and aversive (bottom) stimuli were more extreme the more posterior the contact position. The gradient is shown (solid line) along with the mean normative valence ratings obtained from control subjects (horizontal dashed line), the 95% confidence interval of the mean (dotted line), and the 95% prediction interval of individual normative ratings (sparsely dotted line). The mean valence ratings (circles) are plotted for each patient, averaged over all stimuli and both the DBS conditions. For erotic stimuli, the gradients in both DBS conditions were identical. For valence ratings of aversive stimuli, the slopes of gradients in the DBS OFF and ON conditions were equal, but their offsets differed by 0.17 on the valence scale. Note that the gradients of both aversive and erotic ratings reached the mean normative rating (equality point) nearly at the same position of y = −12.3 mm (vertical dashed line). Note also, that two patients with contacts located posteriorly to this position had erotic valence ratings outside the 95% normative prediction interval, suggesting that the posterior contact position lead to an abnormally exaggerated perception of emotional stimuli. (**b**) The gradient of erotic arousal ratings, identical in both DBS conditions, was similar to the gradient of erotic valences shown in (**a**). The gradient computed from all patients (solid line) and omitting the two patients (#3, #19) who rated the erotic stimuli as very low (dashed line) are shown. Note that one patient with contacts located posteriorly had erotic arousal ratings outside the 95% normative prediction interval, suggesting that the posterior contact position lead to an abnormally exaggerated emotional perception. (**c**) The gradient of aversive arousal ratings, identical in both the DBS conditions, computed from all patients (solid line) and omitting the two outliers (dashed line). Circles marked in red indicate patients who developed depression at long-term follow-up. Note that more posterior electrode placement was associated with a higher occurrence of depression.

The valence ratings of erotic stimuli were gradually affected by the active electrode contact position. The more posterior the contact position, the higher the valence of erotic stimuli (χ^2^(1) = 9.69, P = 0.0019) (Fig. 2a, top). This gradient, defined as the change in affective rating per unit length, did not differ between the DBS conditions (χ^2^(1) = 0.90, P = 0.3431). The equality point, defined as the position along the antero-posterior axis in which patients, on average, rated the valence equally to control subjects, was at y = −12.3 mm for erotic pictures. On average, the valence increased by 0.36 on the valence scale for each 1 mm of contact shift in the posterior direction.

Similarly, valence ratings to aversive stimuli were also affected by the active electrode contact position. The more posterior the contact position, the lower the valence of aversive pictures (χ^2^(1) = 12.7, P = 0.0004) (Fig. 2a, bottom) showing a 0.37 decrease with each 1 mm contact shift in the posterior direction. This gradient also did not differ between the DBS conditions (χ^2^(1) = 1.87, P = 0.1715), and the equality point was similar in both DBS OFF (y = −12.6 mm) and ON (y = −12.2 mm) conditions.

The arousal ratings of erotic stimuli also correlated with the active electrode contact position. The more posterior the contact position, the higher the erotic arousal (χ^2^(1) = 5.43, P = 0.0198) (Fig. 2b). This represents a gradient of 0.60 increase for each 1 mm of contact shift in the posterior direction with the equality point located at y = −11.0 mm. This gradient did not differ between DBS conditions (χ^2^(1) = 1.95, P = 0.3767).

In aversive stimuli, arousal ratings did not seem to correlate with the active electrode contact position (χ^2^(1) = 2.71, P = 0.0998). However, this negative result could be explained by two outlying patients (#3 and #19) who rated the arousal of both erotic and aversive stimuli as relatively low (Fig. 2b,c). Omitting these outliers, an antero-posterior gradient in aversive arousal ratings could be seen as well. The more posterior the contact position, the higher the aversive arousal was (χ^2^(1) = 5.01, P = 0.0251) (Fig. 2b) showing 0.34 increase per 1 mm of contact shift with no difference between the DBS conditions (χ^2^(1) = 0.0034, P = 0.9533) and with the equality point located at y = −8.8 mm.

### Spatial organization of valence and arousal gradients

**Figure 3:**
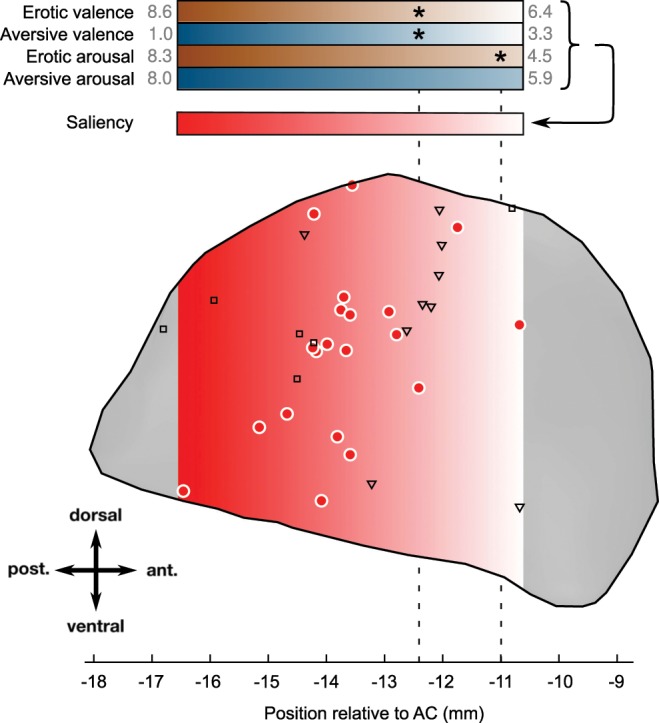
Positions of active electrode contacts and emotion-related neurons in the STN and the emotional gradient therein. Active electrode contacts (denoted by red filled circles) positioned towards the dorsolateral motor part of the STN were found to affect both the valence and arousal ratings of both erotic and aversive stimuli (top four colour gradients, enriched with rating values) along the antero-posterior direction (see also Fig. 2). More posterior contacts resulted in more extreme ratings of emotional pictures. This uniform gradient-like organization can be interpreted as a motivational salience gradient (red-to-white gradient, red indicating higher salience). Asterisks denote equality points, i.e. antero-posterior positions at which the emotional rating equalled the ratings from matched healthy controls. For valence ratings, the equality points were about 12.3 mm posterior to the anterior commissure (vertical dashed line). The gradient is depicted within the region between the most anterior and the most posterior contacts. The positions of previously found emotion-related neurons^15^ responding either to emotional valence or arousal are overlaid (valence-related neurons denoted by squares, arousal-related neurons denoted by triangles). The STN contour corresponds to the sagittal projection of the STN from the Ewert atlas^71^.

The spatial organization of the emotional gradients, i.e. the direction and magnitude of the relative change in the affective ratings per 1 mm in the antero-posterior direction, was similar for the valence ratings from both erotic and aversive stimuli and for arousal ratings from erotic stimuli (Fig. 3). Additionally, the antero-posterior position in which patients rated pictures similar to healthy controls (the equality point) was similar for these emotional dimensions. However, the effect of the electrode position on valence ratings was stronger than on arousal ratings, which is consistent with our previous findings^23^.

The emotional gradients described above were found with respect to the position of the electrode contact computed as the average from the left and right active electrode contacts of the bilateral stimulation. This approach could confound the precise examination of emotional changes. Thus, to assess the potential impact of this averaging procedure, we studied the emotional ratings in respect to the position of the left and right active electrode contacts separately and continued to find similar gradients when considering the left and right contacts separately (Appendix Figs. A1, A2, Table A1).

### Acute effects of subthalamic stimulation: DBS ON vs. OFF comparison

Motor involvement assessed by the motor score of the Unified Parkinson's Disease Rating Scale (UPDRS III) score decreased from 40.2 (SD 10.5) in the DBS OFF condition to 17.8 (6.6) in the DBS ON condition (t(19) = 11.9, P <0.0001). This decrease in UPDRS III did not correlate with the active electrode contact position (F(3,16) = 0.69, P = 0.57) in any direction.

The comparative analysis of DBS ON vs. OFF conditions on emotional assessment showed a significant difference in valence ratings for aversive stimuli only. In the DBS ON condition, patients rated the valence of aversive pictures lower (on average by 0.17, t(19) = −3.21, P = 0.0046) compared to the OFF condition. However, there was no difference in the arousal ratings of aversive stimuli (t(19) = 1.19, P = 0.25). Also, valence and arousal ratings of erotic stimuli did not differ between DBS conditions. Thus, we reproduced our previous results suggesting an increased activation of the aversive motivational system and attributing to a higher salience to aversive stimuli due to STN DBS^23,25^. Interestingly, the size of the DBS-related decrease in the valence ratings of aversive stimuli did not depend on the electrode position. Moreover, this acute DBS effect on aversive valence ratings (i.e., lowering by 0.17) was an order of magnitude weaker compared to the strong chronic effect of the electrode position (i.e., lowering by 2.2, considering the most anterior and the most posterior positions of the active electrode contact).

### Additional analysis

We investigated the possibility that the changes to the affective ratings were related to the surgical intervention itself, i.e. due to a STN lesion caused by the insertion of the DBS electrode and/or its pure presence. We found similar position-related effects even after adjusting for the STN lesion volume. At the same time, the ratings variability could be better explained in terms of the active electrode contact position than the electrode tip position (see Appendix B for details).

### Psychiatric long-term outcome analysis

While only one patient suffered from depression preoperatively, 11 patients developed depressive and/or anxiety disorder during the follow-up period (mean follow-up was 11.1 y (SD 2.7 y, range 5–16 y). The average time from the operation to the diagnosis of depression was 4.7 y (SD 3.7 y). In 3 out of the 4 patients who were diagnosed with depression within the first year after surgery, the onset was clearly related to a drastic reduction of dopaminergic treatment by more than 75%.

Most interestingly, all eight patients with the electrode located within the posterior part of the STN (further than 13.9 mm behind the anterior commissure) had developed depression at the long-term follow-up. On the other hand, only 3 out of the 12 patients with an electrode located more anteriorly developed depression. Notably, the electrode position could predict the occurrence of later depression in our patient sample. On average, the shift in the electrode contact of 1 mm in the posterior direction was associated with increased odds to depression by 390% (χ^2^ = 6.78, P = 0.0092) adjusted for the preoperative BDI score.

In contrast, six patients suffered from a psychotic complication during the follow-up period. Three patients had their electrode located within the posterior part of the STN and three in the anterior part. The electrode position could not predict the occurrence of psychosis (χ^2^ = 2.79, P = 0.0948, adjusting for the preoperative BDI score).

## Discussion

Our results provide evidence for the effects of the electrode position within the motor part the STN on the subjective emotional experience of patients with PD treated with chronic bilateral DBS. Irrespective of whether the DBS was turned ON or OFF, patients with more posterior active electrode contacts reported both valence and arousal ratings from both rewarding and aversive pictures as more extreme than patients with contacts located anteriorly. Therefore, as both the valence and arousal representations seem to be uniformly arranged along the antero-posterior direction and spatially overlap, this gradient-like organization could represent a general code for stimulus motivational salience within the dorsolateral STN^26^.

The electrode position affecting the subjective experience of emotional pictures was within the motor region and was accompanied by excellent effects of DBS on motor symptoms. None of our patients had the active electrode contact placed in the limbic part of the STN which had previously been associated with acute emotion and mood changes^27–30^.This finding further demonstrates the functional overlap of motor and non-motor regions in the STN and supports its key role in emotional processes and integrative function^14,20^.

The electrode position-related effect on the subjective emotional experience was present in both the DBS ON and the OFF conditions which could be a consequence of several mechanisms: (i) surgical intervention (i.e. due to potential damage to the STN parenchyma caused by the insertion of the DBS electrode as already suggested by verbal fluency impairment^31^), or (ii) due to the long-term structural effects of the DBS electrodes within the brain parenchyma (i.e. axonal, glial and reactive inflammatory changes as evidenced by several histopathological autopsy studies on brains of DBS patients^32,33^), or (iii) due to the long-term effects of chronic STN DBS (i.e. persisting after-effect of DBS of the stimulated volume)^34,35^. We found similar position-related effects even after adjusting for the STN lesioned volume, and a weaker position-related effect when considering the tip of the electrode instead of the active contact suggesting that the position of the electrode itself within the STN and/or the long-term effects of chronic STN DBS are more likely to explain our results. DBS induces hierarchical and temporally disparate changes across the brain that in some cases parallel the latencies of improvement in symptoms upon turning the stimulator on and the worsening of symptoms after turning the stimulator off^35^. The lack of differentiation between gradients in the DBS ON and OFF conditions on the one hand and the lack of position-related effects on motor symptoms on the other could also be explained by a hypothetical instant spread of acute stimulation to distances much larger than the range of the active electrode contact positions, which would be manifested in position-non-specific acute effects.

The only change in affective ratings due to the stimulation switched ON was a decrease in valence to aversive pictures which is in agreement with our previous work suggesting that the acute STN DBS drives the affective ratings away from the normative ratings and activates the aversive motivational system^23,25^. However, OFF-medication related distress with a bias towards aversive stimuli could also interfere with the affective ratings. This could potentiate the aversive activation due to the stimulation switched ON while masking some subtle counter-acting DBS induced changes in the subjective experience of the appetitive stimuli. Interestingly, acute changes were relatively weak as compared to the effects of the electrode position within the STN.

**Figure 4:**
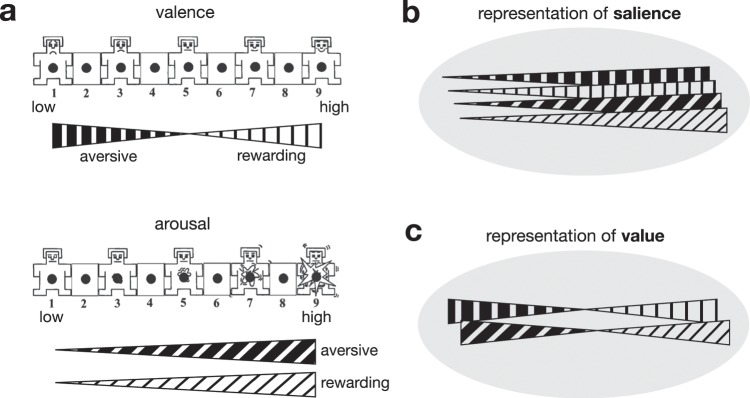
Hypothetic neural representation of value and salience signals based on the organization of the position-dependent gradual changes in emotional ratings. (**a**) Original emotional valence and arousal rating scales images of the SELF ASSESSMENT MANIKIN © Peter J. Lang 1994^65,72,73^. Valence varies from negative (low), to neutral to positive (high), and arousal from low to high. (**b**) Salience signals convey information about the stimulus's significance. The salience is low for non-rewarding and non-aversive stimuli and high for both rewarding and aversive stimuli. Position-dependent gradual change of emotional ratings from neutral valence and low arousal in one part of an anatomical region to extreme (both positive and negative) valence and high arousal in another part is indicative of salience. (**c**) Value signals quantify how good or bad something is. Position-dependent gradual change of valence and arousal from very negative and highly arousing in one part, neutral and low arousing in the middle, and to very positive and highly arousing in another part of an anatomical region is indicative of value.

Two important types of signals relate to emotionally charged stimuli - the value and the salience signals. Each has distinct roles in motivational control and have been linked to distinctive neural circuits^26,36^, which can be differentiated in our study design (Fig. 4). Value signals quantify how good or bad something is, and is important for seeking goals, evaluation of outcomes, value learning, and decision making^26^. On the other hand, salience signals convey information about the stimulus's significance, and are important for orienting attention, arousal, enhancement of cognitive processing, immediate behavioural reaction, and general motivational drive^26^. Salience is low for non-rewarding and non-aversive stimuli and high for both rewarding and aversive stimuli.

In this study, we found a position-dependent gradual change of emotional ratings from moderate valence and low arousal in the anterior part of the motor STN to extreme valence and high arousal in its posterior part, which is indicative of salience representation in the motor region of the STN. Conversely, a representation of the stimulus value would have been represented by a gradual position-dependent change of valence from very negative in one part, neutral in the middle, and very positive in another part of the STN.

Indeed, several lines of evidence from both animal and human studies have indicated the involvement of the STN in salience attribution^20,37,38^. Within the frontal cortical areas, such as the anterior cingulate gyrus and the anterior insula, a number of studies have revealed a distributed neural representation of motivational salience as organized gradients for both rewards and punishments^36,39,40^. Thus, the direct topographically organized and overlapping cortical projections from these regions to the STN can provide the basis for conveying various motivational properties of a stimulus in an organized gradient like-manner to the nucleus for integration and subcortical representation of salience.^14,41^ Within the basal ganglia, the STN is under a strong influence from both the external pallidum, which receives several inputs from limbic structures, such as the amygdala, the paraventricular nucleus of the hypothalamus, the ventral pallidum^41–43^, and midbrain dopaminergic neurons. Dopamine signalling has long been recognized as an essential contributor to coding of a reward-prediction-error and incentive salience processing in the basal ganglia^26,44^.

Following our previous findings of subthalamic affective neurons^45^, this study further extends evidence for a topographic organization of neural maps of the emotional dimensions of valence and arousal within the same anatomical space. While microelectrode recordings identified two spatially segregated neuronal populations responding either to emotional valence or arousal, suggesting their separate processing at a small-scale level, the macroelectrode-related effects demonstrated a smooth gradient-like organization of these two emotional dimensions on a large-scale level within the same anatomical region. Such a two-level organization provides evidence for both segregation and integration of these emotional dimensions within the STN. The interaction between multiple small maps located within larger maps may be complex and may support the emergence of new response properties^46^. Similar to our findings, different large-scale and small-scale organizations have been found in visual and auditory system studies that have often revealed a heterogeneous functional micro-organization embedded in the smooth macroscale order. Topographic maps with different scales recur throughout the nervous system. They seem to play a role in higher-level cognition enabling a rapid and faithful relay of information between the two regions^47^ and as is now suggested by our study, including brain systems coding motivational value and salience. Conceivably, the topographic organization in the STN might overlap with other types of organizations such as convergence or divergence in addition to numerous other forms of connectivity^45^.

However, inferences drawn from the functional anatomy of the STN have to be taken with caution because the study was conducted with PD patients, who are known to suffer from a widespread central nervous system pathology^48^ and experience problems with emotional processing^49^. Furthermore, the presence of the gradient in the STN is not necessarily emotion-specific. It could just be a consequence of selectively increased susceptibility of the posterior part of the STN to the active presence of a DBS electrode with the possible roles of lesion, plasticity, or persisting DBS after-effects. Alternatively, the effects on emotional processing could be mediated by the spread of stimulation to structures posterior to the STN, i.e. the zona incerta^50,51^. However, as compared to the STN DBS no psychiatric side-effects were associated with DBS of the zona incerta^52–55^. Moreover, evidence indicates that stimulation effects are also due to axonal modulation and affection of a fibre network^56^. The hyperdirect cortico-subthalamic fibres from the dorsolateral prefrontal and orbitofrontal cortex and cingulate gyrus and the pallidothalamic fibres are located dorsally and posteriorly to the STN and the observed effects on emotional processing could be related to the stimulation of these tracts^7,57^.

Integrative subthalamic hubs and their projections could be important sites of abnormal long-term plasticity due to DBS and may be linked to long-term psychiatric outcomes in STN DBS treated patients resulting in postoperative depression or psychosis. The frequency of postoperative depression requiring antidepressant treatment is usually high in these patients (up to 60% during the follow-up period of 10 years, similarly to our findings)^20,58^ and the origin seem to be heterogeneous. A reduction of dopaminergic treatment within the first year after surgery^59^ may explain a severe depression only in tree patients in our study. As previously suggested postoperative depression was not found to be related to electrode location during the first year after the STN DBS surgery^19,60^. However, our data suggest that posterior electrode placement within the motor part of the STN might be associated with an increased risk of depression during later periods. In contrast, psychosis frequency was proportionate to the PD duration and did not depend on electrode position which also fit with the hypothesis that the topography found in our study is solely emotional.

As the variability in electrode position was not related to the extent of motor symptom improvement, the more anterior electrode placement in the anterior part of the STN might be associated with good clinical motor outcomes without negative effects on emotion processing and depression development.

## Conclusion

This study on the impact of STN DBS electrode position on emotional experience provides evidence for a causal brain–emotional behaviour relationship. Valence and arousal representations seem to be organized along the antero-posterior direction within the motor part of the STN.

The finding of more extreme ratings from both rewarding and aversive stimuli suggests the attribution of higher emotional salience to all emotional stimuli perceived under DBS in the posterior part of the STN. While there was no impact of the electrode position on motor symptom reduction, more posterior active electrode contacts were associated with more extreme ratings of emotional stimuli compared to control subjects and poorer long-term psychiatric outcomes due to a higher occurrence of depression.

This finding is highly relevant for clinical decisions on electrode placement as well as for the understanding of the mechanisms underlying emotion dysregulation reported in the DBS STN.

## Materials and Methods

### Subjects

Twenty PD patients (all males; mean (SD) age 58.4 (6.0) y, range 45.6–67.0 y; mean disease duration 15.3 (4.4) y), range 9–26 y; treated with bilateral STN DBS for motor fluctuations and/or dyskinesias (mean STN DBS duration 2.2 (1.5) y, range 0.4–6.0 y) and 21 control subjects (all males; mean (SD) age 56.1 (6.5) y, range 39.5–67.1 y); were included in the study. All the patients fulfilled the UK Brain Bank Criteria for the diagnosis of PD^61^.

The study was approved by the local Ethics Committee of the General University Hospital in Prague and all participants gave their informed consent prior to being included in the study. The methods were carried out in accordance with the relevant guidelines and regulations.

On the day of the study all participants were screened for cognitive and mood status using the Mini Mental State Examination (MMSE)^62^ and the Beck Depression inventory (BDI)^63^. The patients’ demographic variables and disease characteristics are summarized in Table 1. No differences were found for age, MMSE, BDI or education duration between the patients and control group. In the PD group, the mean daily dose of dopaminergic medications (in levodopa equivalents^64^ was 684 (SD 532) mg, range 250–2250 mg). Four patients were on long-term antidepressant therapy (1 on citalopram, 1 on mirtazapine, 1 on sertraline, 1 on a combination of escitalopram and mirtazapine). One of the control subjects was on anxiolytic therapy with buspirone. No other psychotropic medication was taken. All patients were chronically stimulated by bilateral monopolar STN DBS. The mean levodopa change was 51% of the preoperative dose (range 18–120%). After the surgery all patients had follow-up visits in our Movement Disorders Centre. We recorded the data on postoperative psychiatric outcomes: (i) the presence of clinical depression that required an initiation of treatment with antidepressants, the year of depression onset, the pre-operative levodopa equivalent, and the levodopa equivalent at the time of depression onset, and (ii) the presence of psychosis, and the year of psychosis onset.

**Table 1:** Parkinson’s disease patients and the control group – demographic and disease characteristics.

		PD Patients	Controls
Age (years)		58.4 (6.0) 45.6–67.0	56.1 (6.5) 39.5–67.1
Education duration (years)		13.4 (2.3) 10–19	16.8 (2.8) 12–23
MMSE		28.7 (1.1) 26–30	29.4 (0.9) 27–30
BDI (preoperative)		7.6 (5.3) 0–22	7.9 (5.4) 2–19
Disease duration (years)		15.3 (4.4) 9–26	n.a.
Time interval after surgery (years)		2.2 (1.5) 0.4–6.0	n.a.
DBS STN Parameters	Frequency (Hz)	130 (0) 130	n.a.
	Pulse width (µs)	75.8 (20.4) 60–120	n.a.
	Amplitude (V)	2.8 (0.4) 2–3.4	n.a.

Values are expressed as means (SD) and ranges.

MMSE, Mini Mental State Examination; BDI, Beck Depression Inventory;

DBS STN, Deep Brain Stimulation of the Subthalamic nucleus; n.a., not applicable.

### Affective task and procedure

Twenty-one pictures representing visual sexual stimuli (erotic females and couples) and 21 aversive pictures representing victims (mutilations) and threat (human or animal attacks, aimed guns) selected from the International Affective Picture system (IAPS)^65^ were rated during the emotional task. The numbers of the selected pictures are presented in the Appendix C1.

Patients were tested after withdrawing from dopaminergic medication overnight. The last medication dose had been administered no later than at 8 p.m. the day before testing, so that the average time the patients were off medication was at least 16.3 hours (min 11.9, max 19.5 h). On the day of testing their stimulators were switched OFF for 2 hours starting at 8 a.m. Then they were tested in the two conditions with the STN DBS switched ON and OFF in counterbalanced orders. There was a 1-hour break between when the stimulators were switched into the particular condition and affective testing (thus the stimulators had been switched OFF for 3 hours in patients who were tested in the OFF condition first). Pictures were presented to PD patients in different orders in the DBS ON and DBS OFF conditions. In each condition prior to affective testing the Unified Parkinson's disease rating scale (UPDRS III) rating was performed by a rater who was unaware of the DBS condition.

Each picture was presented on a touch-sensitive screen for a period of 6 s. As described previously, subjects were required to rate each picture separately along the dimension of emotional valence and arousal on two independent visual scales^25^. Valence was rated on a 1–9 scale, with 1 being the most negative, 5 being neutral, and 9 being the most positive (i.e. lower valence for aversive stimuli means a more aversive/negative assessment). Arousal was rated on a 1–9 scale, with 1 being the calmest and 9 being the most arousing.

### Mapping of the electrode positions

The position of the active electrode contacts were determined as described previously^45^: First, both the frameless preoperative 3.0 Tesla T_2_-weighted (T_2_w) MRI visualizing the STN, and the frameless postoperative 1.5 Tesla T_1_-weighted (T_1_w) MRI displaying the susceptibility artifact of the permanent DBS electrode were automatically co-registered with the frame-based preoperative 1.5 Tesla T_1_w MRI used for neurosurgical planning in each patient using the SurgiPlan software (Elekta, Stockholm, Sweden), thereby getting all preoperative and postoperative images for each patient into individual stereotactic space. The frameless postoperative 1.5 Tesla T_1_w MRI was acquired approximately 2–5 days after DBS implantation (MP-RAGE, i.e. Magnetization-Prepared Rapid Gradient Echo sequence, 160 axial slices with a spatial resolution of 0.9 × 0.9 × 1.65 mm, TR = 2140 ms, TE = 3.93 ms, FA = 15 degrees). The position of the active electrode contact in each STN (defined as x, y, z coordinates in mm) was reconstructed based on the location of the T_1_w MRI susceptibility artifact of the permanent DBS electrode using SurgiPlan software. Then, to get the active contacts from the STN of all patients into one reference space, twelve points delineating anatomically each individual STN and 2 additional points – the anterior commissure (AC) and the posterior commissure (PC) - were identified manually in axial and coronal projections in the preoperative 3 Tesla T_2_w MRI (28 axial slices and 21 coronal slices, 2 mm thick, with x-y resolution 0.9 × 0.9 mm; TR = 2430 ms, TE = 80 ms). The delineating points were co-registered with the 14 corresponding points of the reference model that consisted of coronal slices of the STN manually digitized from the atlas by Morel^24^ using a linear mapping approach that involved shifting, scaling and rotation. In each patient we averaged the positions of the active electrode contacts from both hemispheres as a common electrode contact defined in the STN model as the point lying halfway between the right active electrode contact and the left active electrode contact mirrored to the right hemisphere. The mean distance between such pairs of the active electrode contacts was 2.5 (SD 1.1) mm (range 1.0 to 5.0 mm).

Finally, we performed another linear transformation of the Morel atlas into standardized stereotactic MNI 152 ICBM space^66^ used by the Lead-DBS tool^67^, in which visualization in context of the Ewert atlas^68^ was carried out. We found optimal transformation parameters (shifting, rotation and scaling) that minimized the sum of square distances between all points on the coronal slices of the Morel atlas and the surface of the Ewert atlas. All rotations were below 13° (α_x_ = 0.6°, α_y_ = −11.1°, α_z_ = −13.0°) and scaling was below 30% (s_x_ = 92.9%, s_y_ = 77.7%, s_z_ = 128.4%).

### Statistics

The difference in the UPDRS III score in the ON and OFF conditions was assessed using the Student's paired t-test. The difference in the affective ratings of individual stimuli between DBS ON and OFF conditions was assessed using linear mixed effects models. To capture the repeated nature of ratings specific to patients and stimuli, one random effect was included for each picture, one for each patient in each DBS condition, and one for each combination of picture and patient.

To assess the spatial gradient in the affective ratings, we fitted linear mixed-effects models that attempted to explain the ratings of individual stimuli in terms of the fixed effects of the active electrode contact position along the medio-lateral, antero-posterior, and dorso-ventral directions, DBS condition (ON or OFF), and two covariates to adjust for the patients’ post-operative depressive condition: the BDI mood status and the indication of any anti-depressive therapy (which was uncorrelated with the mood status). Random effects were included for each picture, for each patient in each DBS condition, and for each picture-patient pair. To identify the simplest, yet well-fitting model, we successively eliminated individual spatial directions from the models and stopped when the P-value of the model-submodel likelihood ratio test was less than 0.1, which suggested that the submodel, having the direction removed, could not fit the data as well as the model including the direction. To assess the spatial gradient in motor symptom reduction, we related the DBS OFF-ON difference in the UPDRS III score to the three spatial directions using a linear model. To assess the influence of the active contact position on the postoperative psychiatric outcomes, we used a logistic regression model with an additional covariate of preoperative BDI to adjust for the preoperative mood status. Statistical analyses were carried out in R^69^.

## Supplementary information

Supplementary information

## Data Availability

The datasets generated during and/or analysed during the current study are available from the corresponding author on reasonable request.
