# Supplementary material for: Topography of emotional valence and arousal within the motor part of the subthalamic nucleus in Parkinson’s disease
Source: Sci Rep. 2019 Dec 27;9:19924. doi: 10.1038/s41598-019-56260-x (PMC6934686; doi:10.1038/s41598-019-56260-x)
Supplement: Supplementary file 1 — Supplementary information [file 41598_2019_56260_MOESM1_ESM.pdf]

Topography of emotional valence and arousal within the motor part of the subthalamic nucleus in Parkinson's disease

Short title: Emotion topography in the subthalamic nucleus

Tereza Serranová[1*†], Tomáš Sieger[1,2*], Filip Růžička[1,3], Eduard Bakštein[2,4], Petr Dušek[1], Pavel Vostatek[2], Daniel Novák[2], Evžen Růžička[1], Dušan Urgošík[3], Robert Jech[1,3]

* authors contributed equally to this work

[1] Dept. of Neurology and Center of Clinical Neuroscience, Charles University in Prague, 1st Faculty of Medicine and General University Hospital, Kateřinská 30, 128 08, Prague, Czech Republic

[2] Dept. of Cybernetics, Faculty of Electrical Engineering, Czech Technical University, Technická 2, 166 27, Prague, Czech Republic

[3] Dept. of Stereotactic and Radiation Neurosurgery, Na Homolce Hospital, Roentgenova 2, 150 30, Prague, Czech Republic

[4] National Institute of Mental Health, Klecany, Topolová 748, 250 67, Czech Republic

Appendix A

Table A1.

The gradients of emotional ratings in respect to the antero-posterior position of the left and right active electrode contacts.

	Left contacts				Right contacts			
	Valence ratings		Arousal ratings		Valence ratings		Arousal ratings	
	Erotic	Aversive	Erotic	Aversive †	Erotic	Aversive	Erotic	Aversive †
Rating change per 1 mm	0.30	-0.31	0.63	n.a.	0.32	-0.32	0.46	0.32
Gradient significance	$\chi^2(1)$ = 6.62, P = 0.0101	$\chi^2(1)$ = 9.00, P = 0.0027	$\chi^2(1)$ = 5.49, P = 0.0192	$\chi^2(1)$ = 3.01, P = 0.0828	$\chi^2(1)$ = 9.17, P = 0.0025	$\chi^2(1)$ = 11.08, P = 0.0009	$\chi^2(1)$ = 4.14, P = 0.0419	$\chi^2(1)$ = 5.56, P = 0.0183
Equality point (mm)	-12.1	-12.2	-11.2	n.a.	-12.1	-12.1	-10.2	-8.3

Gradients in affective ratings are expressed in terms of a change of rating per 1mm (when significant), the gradient significance (χ^2 statistics and P-value of the model-submodel test), and the equality point, defined as the position along the antero-posterior axis in which patients, on average, rated the stimuli equally to control subjects. No gradient differed between DBS conditions.

† Omitting outlying patients (#3 and #19) who rated the arousal of both the erotic and aversive stimuli relatively low in respect to other patients and controls. n.a., not applicable.

Fig. A1

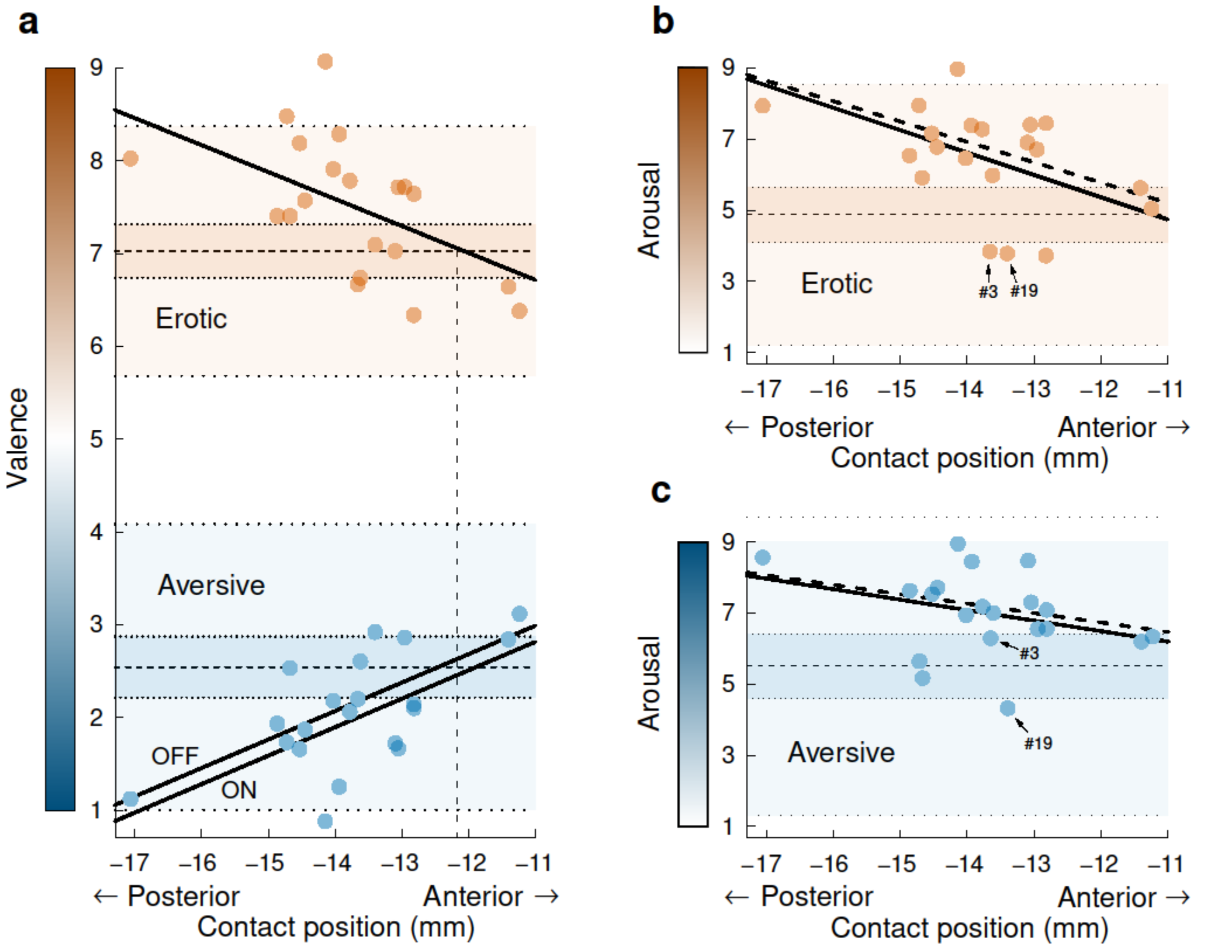

The gradients of emotional ratings in respect to the antero-posterior position of the active contact on the left electrode.

(**a**) Valence ratings of erotic (top) and aversive (bottom) stimuli were more extreme the more posterior the contact position. The gradient is shown (solid line) along with the mean normative valence ratings obtained from control subjects (horizontal dashed line), the 95% confidence interval of the mean (dotted line), and the 95% prediction interval of individual normative ratings (sparsely dotted line). For erotic stimuli, the gradients in both DBS conditions were identical. For valence ratings of aversive stimuli, the slopes of gradients in the DBS OFF and ON conditions were equal, but their offsets differed by 0.17 on the valence scale. Note that the gradient of both aversive and erotic ratings reached the mean normative rating (equality point) at the same position of about y=-12.1 mm. (**b**) The gradient of erotic arousal ratings, identical in both DBS conditions, was similar to the gradient of valences shown in (**a**). The gradient computed from all patients (solid line) and omitting two patients (#3, #19) who rated the erotic stimuli very low (dashed line) are shown. (**c**) The gradient of aversive arousal ratings, identical in both the DBS conditions, computed from all patients (solid line) and omitting the two outliers (dashed line).

Fig. A2

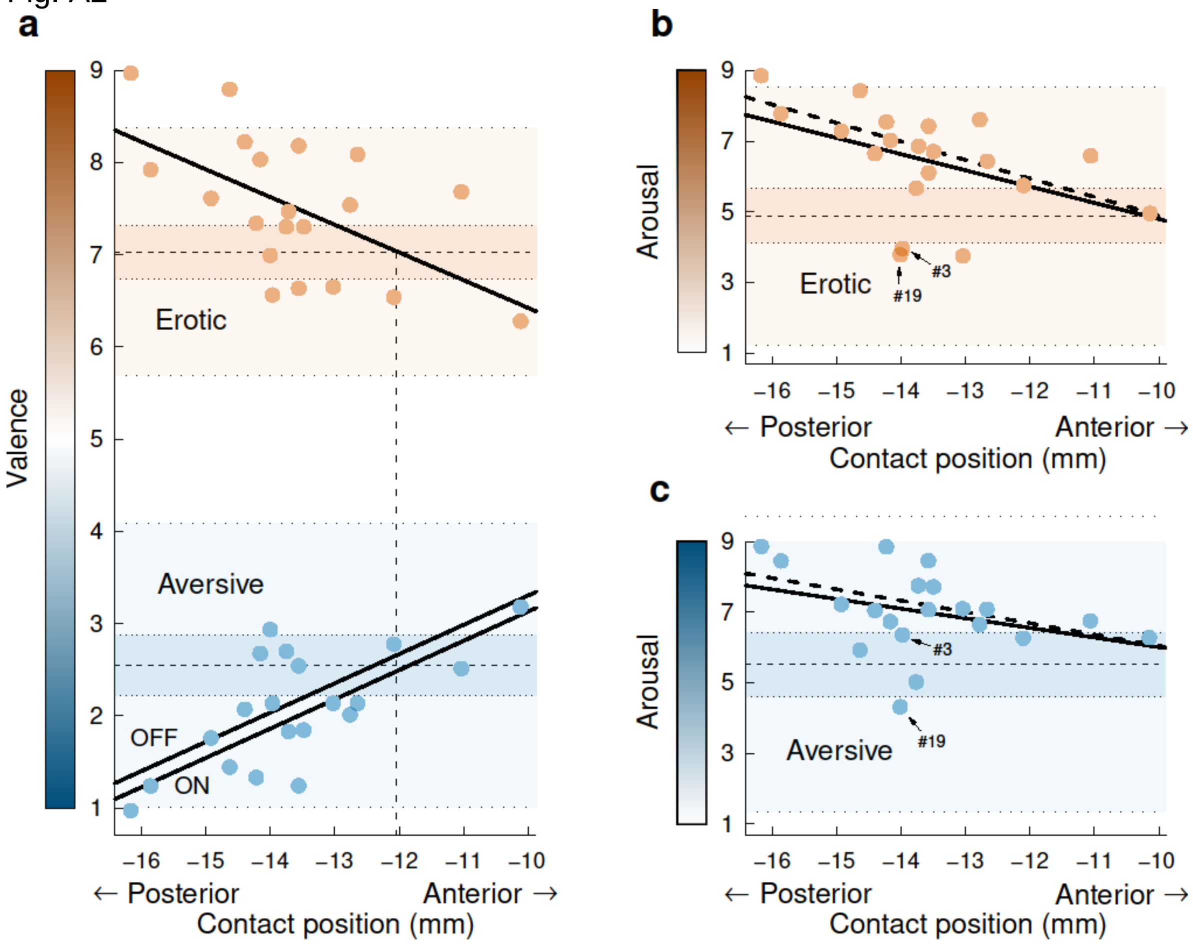

The gradients of emotional ratings in respect to the antero-posterior position of the active contact on the right electrode.

The legend is the same as for Fig. A1.

Appendix B

Can the position-related emotional effects be explained by micro-lesion?

As the DBS electrode insertion causes micro-lesion of the STN, we asked whether the observed variability on the topographic gradients in the emotional ratings could not be explained by the micro-lesions in the STN rather than the position of the active DBS electrode contact.

We quantified STN micro-lesions in terms of the volume of STN motor, associative, and limbic territories impacted by the DBS electrode. The lesioned volume of each territory on each side was estimated by projecting a simple cylindrical model of the DBS electrode into the standardized atlas of the three territories[70], calculating the number of voxels of each territory intersected by the electrode, and converting this number to the lesioned volume measured in mm^3. Finally, the lesioned volumes from the left and right side were summed. We then included the lesioned volumes as additional covariates to the statistical models of the emotional gradients. If, hypothetically, the gradients were caused by the micro-lesion in e.g. the limbic part of the STN, the topographic gradient would have vanished from the model after adjusting to the micro-lesion.

When adjusted for the micro-lesion, the valence ratings to aversive stimuli were still strongly affected by the active electrode contact position. The more posterior the contact position, the lower the valence for aversive pictures ($\chi^2(1) = 11.43$, $P = 0.0007$), showing 0.36 decrease on the valence scale with each 1 mm contact shift in the posterior direction. Similarly, the valence ratings of erotic stimuli were also gradually affected by the active electrode contact position, even though not purely in the posterior, but rather the postero-lateral direction. The more postero-lateral the contact position, the higher the valence of erotic stimuli ($\chi^2(1)=11.65$, $P=0.0006$), showing 0.82 increase for each 1 mm of contact shift.

The arousal ratings of aversive stimuli also still correlated with the active electrode contact position after adjusting for the micro-lesion. The ratings changed in the postero-ventro-lateral direction, in which a shift of 1mm was associated with 0.43 increase on the arousal scale ($\chi^2(1)=8.67$, $P=0.0032$). In erotic stimuli, the arousal ratings also changed in the postero-ventro-lateral direction, in which a shift of 1mm was associated with an increase of 1.0 ($\chi^2(1) = 10.55$, $P = 0.0012$).

In conclusion, the emotional gradients in the antero-posterior directions (as described in Results) could simply not be attributed to the effects of the DBS-induced micro-lesion.

Can the variability in affective ratings be explained by the pure presence of the DBS electrode?

In the Results section, we described how the valence and arousal ratings gradually changed with the position of the active contact of DBS electrodes. Surprisingly, we did not find a difference in the strength of these gradients between the DBS ON and OFF conditions, which insinuated that chronic rather than acute effects could be associated with the ratings changes. We thus further asked about the nature of these gradients. Could the gradual rating changes be best explained by the position of the active contacts, or rather by the position of the DBS electrode per se?

To answer this question, we built alternative models of valence and arousal ratings in terms of the position of the tip of the DBS electrodes (instead of the position of the active electrode contact). We speculated that if the gradual changes in the ratings were associated with the presence of the DBS electrode regardless of which electrode contact was active, we would have observed much stronger gradients in terms of the electrode tip. If, on the other hand, the gradual emotional changes were implied by DBS delivered through the active contact, a stronger gradient would have been observed considering the active electrode contacts.

The original and the alternative gradients are given in Table B1, where the strength of each gradient and the quality of the corresponding model fit are compared. It is evident that both the strength of the gradient (i.e. the change in rating associated with 1 mm change in the electrode position) and the fit quality were higher for models that explained the ratings in terms of the active electrode contact. We thus concluded that the changes in the emotional experience were caused by the DBS delivered through the active contact rather than by the pure presence of the DBS electrode within the STN.

Table B1.

The gradients of the valence and arousal ratings in terms of the DBS active electrode contact and the tip of the DBS electrode.

	Active contacts				Electrode tip			
	Valence ratings		Arousal ratings		Valence ratings		Arousal ratings	
	Erotic	Aversive	Erotic	Aversive †	Erotic	Aversive	Erotic	Aversive †
Rating change per 1 mm	0.36	-0.37	0.60	0.34	0.33	-0.29	0.50	0.19
Gradient significance (P-value)	0.0019	0.0004	0.0198	0.0251	0.0075	0.0068	0.0584	0.2312
AIC	1978.7	2087.0	2752.5	2795.9	1981.3	2092.4	2754.2	2799.5

Gradients in affective ratings are expressed in terms of a change in rating per 1 mm, the gradient significance (the P-value of the model-submodel test), and the Akaike information criterion (AIC) expressing the quality of the model fit (better fits lead to smaller AIC values).

† Omitting outlying patients (#3 and #19) who rated the arousal of both the erotic and aversive stimuli relatively low in respect to other patients and controls (see Results).

Appendix C

C1.

The numbers of rewarding and aversive IAPS pictures presented in the study

Rewarding (erotic) pictures 4002, 4275, 4320, 4232, 4694, 4180, 4250, 4150, 4240, 4255, 4670, 4235, 4310, 4225, 4311, 4220, 4006, 4659, 4141, 4001, 4142.

Aversive pictures: threats: 1050, 1120, 1300, 3500, 3530, 6230, 6260, 6350, 6510, 6550, victims: 3000, 3010, 3060, 3069, 3071, 3080, 3120, 3130, 3170, 3266, and threat/victim picture 9410.
